# *In vivo* assessment of increased oxidation of branched-chain amino acids in glioblastoma

**DOI:** 10.1038/s41598-018-37390-0

**Published:** 2019-01-23

**Authors:** Eul Hyun Suh, Edward P. Hackett, R. Max Wynn, David T. Chuang, Bo Zhang, Weibo Luo, A. Dean Sherry, Jae Mo Park

**Affiliations:** 1 Advanced Imaging Research Center, UT Southwestern Medical Center, Dallas, TX USA; 2 Department of Biochemistry, UT Southwestern Medical Center, Dallas, TX USA; 3 Department of Internal Medicine, UT Southwestern Medical Center, Dallas, TX USA; 4 Department of Pathology, UT Southwestern Medical Center, Dallas, TX USA; 5 Department of Pharmacology, UT Southwestern Medical Center, Dallas, TX USA; 6 Department of Chemistry and Biochemistry, UT Dallas, Richardson, TX USA; 7 Department of Radiology, UT Southwestern Medical Center, Dallas, TX USA; 8 Department of Electrical Engineering, UT Dallas, Richardson, TX USA

## Abstract

Altered branched-chain amino acids (BCAAs) metabolism is a distinctive feature of various cancers and plays an important role in sustaining tumor proliferation and aggressiveness. Despite the therapeutic and diagnostic potentials, the role of BCAA metabolism in cancer and the activities of associated enzymes remain unclear. Due to its pivotal role in BCAA metabolism and rapid cellular transport, hyperpolarized ^13^C-labeled α-ketoisocaproate (KIC), the α-keto acid corresponding to leucine, can assess both BCAA aminotransferase (BCAT) and branched-chain α-keto acid dehydrogenase complex (BCKDC) activities via production of [1-^13^C]leucine or ^13^CO_2_ (and thus H^13^CO_3_^−^), respectively. Here, we investigated BCAA metabolism of F98 rat glioma model *in vivo* using hyperpolarized ^13^C-KIC. In tumor regions, we observed a decrease in ^13^C-leucine production from injected hyperpolarized ^13^C-KIC via BCAT compared to the contralateral normal-appearing brain, and an increase in H^13^CO_3_^−^, a catabolic product of KIC through the mitochondrial BCKDC. A parallel *ex vivo* ^13^C NMR isotopomer analysis following steady-state infusion of [U-^13^C]leucine to glioma-bearing rats verified the increased oxidation of leucine in glioma tissue. Both the *in vivo* hyperpolarized KIC imaging and the leucine infusion study indicate that KIC catabolism is upregulated through BCAT/BCKDC and further oxidized via the citric acid cycle in F98 glioma.

## Introduction

In tumors, energy dependence on substrates is significantly altered to compensate elevated biosynthesis and bioenergetics needed for tumor proliferation^1^. For instance, most cancer cells rely on energy-inefficient aerobic glycolysis, known as ‘the Warburg effect’^2^, to facilitate the uptake and incorporation of nutrients into the biomass for rapid proliferation. The biosynthetic demands in cancer also stimulate utilization of other nutrients such as glutamine^3^, acetate^4,5^, and branched-chain amino acids (BCAAs: leucine, valine, and isoleucine). In particular, BCAAs are reportedly essential for tumor proliferation^6–8^ and altered BCAA utilization has been demonstrated in multiple cancers^9^. Overexpressed branched-chain amino acid aminotransferase 1 (BCAT1), a cytosolic enzyme that transfers α-amino groups to α-ketoglutarate to yield the respective branched-chain α-keto acid (BCKA), has been suggested as a prognostic cancer indicator^6,10–12^. Moreover, a recent study showed increased expression of mitochondrial BCAT (BCAT2) in pancreatic adenocarcinoma^13^. Despite the emerging roles of BCAAs, however, the molecular mechanism of associated enzyme activities in cancer still remains elusive^8,9,14^.

**Figure 1:**
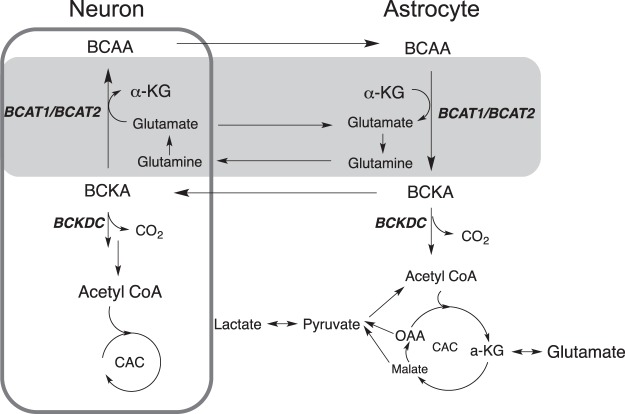
Schematic diagram of BCAA metabolism in the brain. BCAT, branched-chain amino acids aminotransferase; BCKDC, branched-chain α-keto acid dehydrogenase complex; CAC, citric acid cycle; α-KG, α-ketoglutarate; OAA, oxaloacetate.

In the brain, BCAAs serve as nitrogen donors in the glutamine/glutamate cycle as well as sources for energetic and biosynthetic demands^15,16^ (Fig. 1). As the first step of BCAA catabolism, BCAAs are transaminated to BCKAs by BCAT isoenzymes. Although the BCATs catalyze reversible transamination reaction between BCAA and BCKA, the catabolic pathway of BCAAs is the predominant direction in most cell types^15^. α-ketoglutarate is converted to glutamate via BCAT1 in neurons and via BCAT2 in astrocytes to yield the major excitatory neurotransmitter of central nervous system^17^. In the tripartite synapse, astrocytes convert excess glutamate to α-ketoglutarate by reanimating BCKAs to maintain the concentration of glutamate below 5–10 mM as a glutamate-buffering mechanism for brain nitrogen homeostasis. As a result of this regulatory mechanism, for instance, physiological concentration of α-ketoisocaproic acid (KIC) is maintained at 0.7 ± 0.1 μM in rat brain, which is significantly lower than KIC in rat blood plasma (18.9 ± 1.4  μM)^18^. Subsequently, the BCKAs are converted to branched-chain acyl-CoA’s via branched-chain α-keto acid dehydrogenase complex (BCKDC) at an irreversible oxidative decarboxylation step. Thus, CO_2_ produced in this step can serve as a direct indicator of BCAA oxidation. The ensuing enzymatic reactions are unique for each BCAA with end products from each degradative pathway eventually being oxidized through the citric acid cycle (CAC).

In brain malignancies (e.g., glioma), BCAA uptake via L–amino acid transporter-1 (LAT1) is enhanced compared to normal astrocytes^19^ and BCAT1 activity is augmented in primary gliomas with wild-type isocitrate dehydrogenase (IDH^wt^). In particular, the BCATs are suggested as cancer-specific biomarkers, but the directionality of the BCAT-mediated reaction and how the BCAT enzyme levels are associated with metabolic phenotypes in tumor have been addressed in different manners^9^. In glioblastoma, BCAA catabolism is upregulated to support cell proliferation^6^. In contrast, increased reverse direction was observed in non-small cell lung cancer for tissue protein synthesis^14^ and in leukemia for cancer progression^8^. As the tumor metabolism is progressively remodeled by adapting to the surrounding tumor microenvironment, it is important to understand the environmental factors and tumor heterogeneities, and therefore necessary to assess comprehensive tumor characteristics *in vivo*^20^.

Non-invasive assessment of *in vivo* metabolism is crucial to understand the altered BCAA metabolism and to identify potential imaging biomarkers in cancer. Dynamic nuclear polarization (DNP or ‘hyperpolarization’) in combination with a rapid dissolution and ^13^C MR spectroscopy allows *in vivo* investigation of multiple metabolic pathways by detecting metabolic flux of ^13^C labeled substrates^21^. It has been a useful imaging tool for assessing cancer-specific enzyme-catalyzed metabolic phenotypes^22,23^. In particular, hyperpolarized (HP) [1-^13^C]KIC was used to assess BCAT-mediated leucine metabolism in the brain *in vivo*^24^. [1-^13^C]KIC has a relatively long longitudinal relaxation time (T_1_), a key parameter that determines the length of the observable time-window for *in vivo* metabolism. KIC is rapidly transported into the cell via monocarboxylic transporter (MCT)^24^ and can assess both BCAT and BCKDC activities via production of [1-^13^C]leucine or ^13^CO_2_ (and thus H^13^CO_3_^−^), respectively. Indeed, previous studies showed that the HP [1-^13^C]KIC conversion to [1-^13^C]leucine was correlated with BCAT activity in prostate cancer cells, lymphoma, and normal rat brain. In the prostate cancer cells, elevated BCAT levels resulted in increased production of HP [1-^13^C]leucine from HP [1-^13^C]KIC^25^. Another HP [1-^13^C]KIC study observed increased [1-^13^C]leucine conversion in EL4 lymphoma^26^. Moreover, Butt, *et al*. demonstrated that ^13^C metabolic imaging using HP ^13^C KIC is a practical method to access the BCAT activity *in vivo* in rat brains^24^.

We hypothesized that BCAA oxidation would be increased in glioma-bearing rats as a unique feature of tumor, which can be visualized by ^13^C MRI in combination with DNP. In this study, we investigated the increase of *in vivo* leucine oxidation in F98 glioma and the feasibility of HP [1-^13^C]KIC as an imaging agent to measure altered BCAA metabolism in glioma by assessing BCAT- and BCKDC-catalyzed *in vivo* enzyme reactions. Moreover, ^13^C NMR isotopomer analysis was performed after a steady-state infusion of [U-^13^C]leucine into glioma-bearing rats to elucidate the downstream metabolism of BCAA. These results were further evaluated by subsequent *ex vivo* tissue analysis of BCAT/BCKDC enzyme activities and its expression levels.

## Results and Discussion

### GBM animal model

We used F98 cell line for glioma model. It is histologically classified as an anaplastic glioma, which is pathologically undifferentiated malignant glioma^27,28^, and the characteristics of tumor growth are similar with human glioblastoma multiforme (GBM) in overexpression of the epidermal growth factor receptor (EGFR)^28^. The growth of tumors was confirmed 15–18 days after the cell implantation using both T_2_-weighted and contrast-enhanced (CE) T_1_-weighted proton MRI. The tumor regions identified by postmortem hematoxylin and eosin (H&E) stain was better matched to the hyperintense regions of the CE T_1_-weighted-MRI rather than those of T_2_-weighted images (Fig. S1).

### *In vivo*^13^C imaging of branched-chain amino acid metabolism in glioma using hyperpolarized [1-^13^C] α-ketoisocaproate to assess BCAT/BCKDC activity of glioma

**Figure 2:**
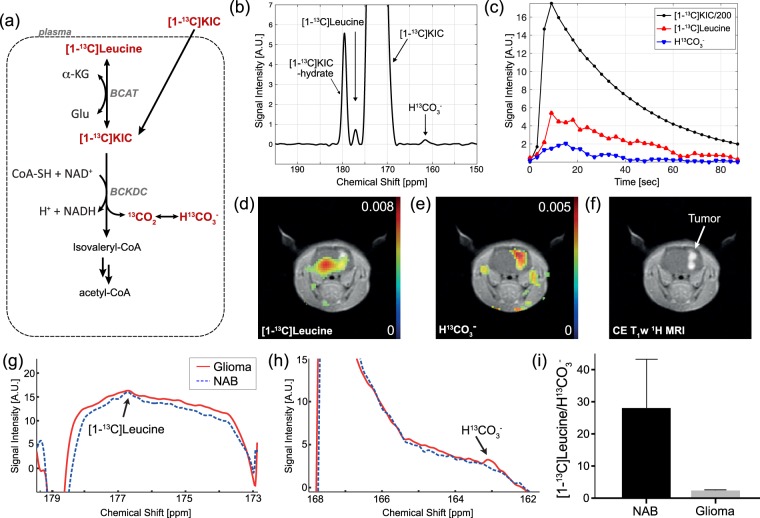
(**a**) Metabolic pathway of hyperpolarized (HP) [1-^13^C]KIC. ^13^C-labeled KIC and its metabolic products are highlighted in red. (**b**) An *in vitro* time-averaged spectrum from F98 cells showed the injected HP [1-^13^C]KIC and produced [1-^13^C]leucine and H^13^CO_3_^−^ peaks. (**c**) The corresponding time courses of HP KIC and the products. (**d**–**e**) *In vivo* chemical shift imaging of a F98 glioma-bearing rat using HP [1-^13^C]KIC and (**f**) the contrast-enhanced (CE) T_1_-weighted ^1^H image. Metabolite distributions of [1-^13^C]leucine and H^13^CO_3_^−^ in a tumor-bearing rat brain slice after an injection of HP [1-^13^C]KIC. (**g**–**h**) The reconstructed spectra in the glioma (solid red) and the contralateral normal-appearing brain (NAB; dotted blue) and (**i**) *in vivo* metabolite ratio of [1-^13^C]leucine to H^13^CO_3_^−^.

We first tested HP [1-^13^C]KIC in F98 glioma cells *in vitro*. A time-resolved pulse-and-acquire ^13^C sequence with a non-selective radiofrequency (RF) pulse was used to monitor the injected [1-^13^C]KIC (δ_13C_ = 172.6 ppm) and products every 3 sec. BCAT and BCKDC activities were detected by the appearance of [1-^13^C]leucine (δ_13C_ = 176.8 ppm) and H^13^CO_3_^−^ (δ_13C_ = 163.1 ppm), respectively. The signal of HP [1-^13^C]leucine and H^13^CO_3_^−^ reached a maximum about 10–15 sec after the injection of HP [1-^13^C]KIC (Fig. 2a–c). All the KIC samples were polarized by a SPINlab™ DNP polarizer. The polarization level and the T_1_ at 3 T were measured as 24% (35 sec after the dissolution) and 77 sec from independent calibration experiments.

For *in vivo* imaging, a two-dimensional spiral chemical shift imaging pulse sequence with a variable RF scheme was used to acquire single time-point metabolite maps of glioma-bearing rat brain following an intravenous injection of HP [1-^13^C]KIC (injection-to-scan = 30 sec, scan time = 0.5 sec)^29^. Upon intravenous bolus injection of [1-^13^C]KIC into F98 glioma rats, [1-^13^C]leucine and H^13^CO_3_^−^ were detected in the rat brain tissue (Fig. 2d–f). Each metabolite was quantified by integrating the corresponding peak in the absorption mode after spectral tomosynthesis^30^ and phase correction. Figure 2g–h are the reconstructed spectra from the tumor and the normal-appearing brain (NAB) for leucine and bicarbonate (HCO_3_^−^), respectively. KIC has been found to cross the blood-brain barrier (BBB) predominantly by a carrier-mediated transport system and subsidiary by simple diffusion under physiological condition^31^. Exogenously injected HP KIC was taken up by MCTs and metabolized to leucine and HCO_3_^−^ by BCAT and BCKDC, respectively^32^. Reversible BCAT reaction enables a carbon isotope exchange between KIC and leucine in addition to the net flux of HP ^13^C from KIC to leucine^33^. Therefore, HP ^13^C KIC to leucine conversion is determined by endogenous ^12^C leucine pool size as well as membrane transport of KIC, delivery to the tumor, and BCAT enzyme activities. On the other hand, conversion of HP KIC to HCO_3_^−^, a decarboxylated product of [1-^13^C]KIC, is irreversibly mediated by BCKDC and, therefore, indicates a pure metabolic catabolic flux. ^13^C-leucine, the transamination product of KIC was smaller in the tumor as compared to the contralateral NAB ([1-^13^C]leucine/total ^13^C = 0.0053 in tumor *vs*. 0.0081 in NAB, p < 0.05). The low ^13^C conversion from KIC to leucine in the tumor implies predominant net conversion of leucine to KIC or suppressed isotope exchange between KIC and leucine (e.g., depletion of intrinsic leucine pool). Conversely, more H^13^CO_3_^−^ was detected in the tumor (H^13^CO_3_^−^/total ^13^C = 0.005, p < 0.05) compared to the NAB (0.0027), suggesting elevated KIC catabolism by BCKDC towards isovaleryl-CoA.

### Increased of [U-^13^C]leucine oxidation in F98 glioma

**Figure 3:**
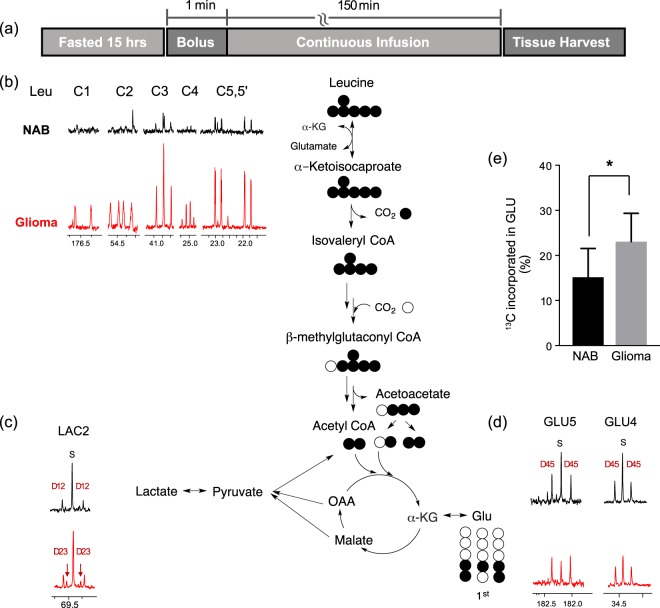
^13^C NMR spectra acquired from glioma and contralateral normal-appearing brain (NAB) *ex vivo* after a steady-state [U-^13^C]leucine infusion. (**a**) Protocol for the [U-^13^C]leucine infusion. (**b**) Elevated leucine uptake and significantly higher (**c**) lactate and (**d**) glutamine labeling were observed in the tumor as compared to NAB, indicating an increased oxidation of leucine in the tumor. (**e**) ^13^C-labeled [4-^13^C]glutamate derived from [U-^13^C]leucine relative to the total glutamate pool size (%) from the leucine infusion study (*p < 0.05). D indicates doublet.

BCAA can be partially oxidized for protein synthesis or can be fully oxidized as an energy source. Although major BCAA oxidative capacity is high in skeletal muscle or liver, brain has 20% proportion of the whole-body capacity to oxidize BCAA^34^ and this rate is greater than incorporation rate of BCAA into protein^15^. Previous studies have shown increased uptake of BCAA by C6 glioma cell^19^ and breast cancer cell lines (MDA-MB-231, MCF-7) via LAT1^35^. To elucidate the metabolic fate of BCAA in glioma, leucine metabolism was further explored in F98 glioma using an *ex vivo* ^13^C NMR isotopomer analysis. The isotopomer analysis can track down multiple metabolic pathways by accessing the distribution of ^13^C isotopomers in tissue after infusion of ^13^C-labeled substrates. We evaluated downstream metabolism of leucine in brain tumor after a [U-^13^C]leucine infusion into F98 glioma-bearing rats. Oxidation of [U-^13^C]leucine in the glioma produced distinct labeling in several CAC-intermediates as downstream products. In particular, ^13^C-glutamate labeling patterns enable direct comparison of leucine utilization between glioma and NAB. The glutamate doublet in ^13^C NMR spectra were also analyzed to determine the quantity of ^13^C labeled acetyl-CoA entering the CAC. The protocol for ^13^C-leucine infusion study is described in Fig. 3a. Both glioma and contralateral NAB tissues were harvested and freeze-clamped after a steady-state infusion of [U-^13^C]L-leucine. ^13^C NMR spectra from glioma and NAB tissue extracts were compared by examining the distribution of ^13^C isotopomers. The data showed that [U-^13^C]leucine was converted to [U-^13^C]KIC and further catabolized to [1,2,3,4,5-^13^C_5_]isovaleryl-CoA and H^13^CO_3_^−^ via mitochondrial BCKDC, followed by conversion to [2,3,4-^13^C_3_]acetoacetate and [1,2-^13^C_2_]acetyl-CoA. Both [1,2-^13^C_2_] and [2-^13^C]acetyl-CoA are oxidized in the CAC and form various ^13^C isotopomers in ^13^C-glutamate. ^13^C NMR spectra reflected higher quantities of ^13^C-labeled leucine in glioma tissue versus NAB tissue (Fig. 3b). Glutamate derived from leucine was estimated from C4 doublet of [4,5-^13^C_2_]glutamate (23.1 ± 6.2% of total glutamate pool in glioma, 15.5 ± 5.9% in NAB, p < 0.05, Fig. 3d,e). Moreover, the multiplets appearing in the lactate C2 resonance were more abundant in the spectrum of glioma tissue, reflecting oxidation of leucine in the CAC followed by cycling of three carbon units out of the CAC and into pyruvate (pyruvate recycling) (Fig. 3c). Based on the ^1^H MR spectroscopy, leucine concentration in brain tissue was significantly higher in glioma (0.278 ± 0.035 μmol/g of wet tissue) than in contralateral NAB (0.075 ± 0.001 μmol/g). Lactate pool was also larger in the glioma (9.989 ± 1.413 μmol/g) compared to the NAB (7.639 ± 1.506 μmol/g) (Fig. S2). Together, these results indicate that leucine uptake and oxidation are increased in glioma. The total leucine plasma level was within the physiological condition during the infusion (91.7 ± 16 μmol/L in tumor-bearing rat)^36^. Increased catabolism of [U-^13^C]leucine in tumor tissue is consistent with the *in vivo* ^13^C imaging results using HP [1-^13^C]KIC in F98 glioma that showed higher decarboxylation product of HCO_3_^−^ from [1-^13^C]KIC in the tumor compared to NAB.

### *Ex vivo* assay of BCAT and BCKDC activities in brain tissues

**Figure 4:**
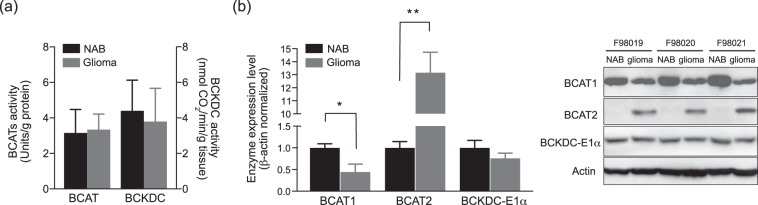
*Ex vivo* assay of (**a**) BCAT and BCKDC activities in brain tissues. (**b**) Protein expression levels, normalized to the protein expression in the contralateral normal-appearing brain (NAB), of BCAT1, BCAT2 and BCKDC-E1α (Western blot analysis, *p < 0.05, **p < 0.01).

To verify the results of the *in vivo* imaging and the steady-state infusion study, enzyme activities and expression levels of BCAT and BCKDC were measured in brain tissues. BCAT activity assays were performed as previously described using *in vitro* continuous spectrophotometric method. L-alanine aminotransferase and L-lactate dehydrogenase were used as coupled enzyme in this assay^37^. BCKDC subunit activity was assayed in the brain tissue according to the methods of radiochemical assay by [1-^14^C]KIC substrate and measure the [^14^C]-decarboxylation by BCKDC^38^. The previous study reported that BCAT1 is a major isoenzyme in rat brain responsible for 60 - 70% of total BCAT activity^39^. This is congruent with our western blot analysis; The expression of BCAT1, rather than BCAT2, is relatively predominant in both NAB and glioma. While the total BCAT activities were comparable between glioma (3.34 ± 0.36 U/gram of protein) and NAB (3.16 ± 0.05), the expression levels of isoenzyme BCAT1 and BCAT2 were significantly tumor-specific: lower BCAT1 and higher BCAT2 in glioma than in NAB (Fig. 4). The distinct levels of BCAT1 and 2 in glioma might be due to the characteristics of its cellular origin, glial cell^28,40^, as tumors conserve the metabolic phenotype of their tissue origin^41^. Indeed, BCAT2 is predominant in glial cells whereas BCAT1 is primarily in cortical neurons^17,42,43^. In contrast, both the activity and the expression level of the BCKDC-E1α subunit were at similar levels in glioma (3.80 ± 1.87 nmol CO_2_/min/g tissue) and NAB (4.41 ± 0.86) (Fig. 4). Increased expression level of BCAT2 and unaltered BCKDC level in glioma together suggests that BCAT2 is a key enzymatic step that regulates BCAA catabolism in glioma. Islam, M.M., *et al*.^39^ recently reported that BCAT2 forms a protein-protein complex with BCKDC such that the substrates like KIC are channeled to the adjacent E1 subunit of BCKDC for further catabolism. Such channeling of substrates could explain the enhanced H^13^CO_3_^−^ production observed in glioma versus normal brain tissue.

### Outlook

GBM is one of the most aggressive primary brain tumors. Nevertheless, diagnosis and prognosis is limited by poor understanding of molecular biomarker^44^ and biopsy is restricted if the tumor is inoperable. Although the role of BCAA in cancer needs further investigation, multiple enzymatic steps of BCAA metabolism can be important prognosis and diagnosis biomarkers in tumor. In particular, BCAT1 expression is dependent on the concentration of α-ketoglutarate substrate in glioma cell lines and could be suppressed by ectopic overexpression of mutant IDH isoform 1 (IDH1) in immortalized human astrocytes, providing a link between IDH1 function and BCAT1 expression. This link is supported by IDH^wt^ astrocytic gliomas being characterized by high BCAT1 expression^6^. The overexpression of mutant IDH1 associated with reduced BCAT1 expression and reduced invasiveness and tumor growth provides a plausible rationale for the aggressiveness of primary GBM and the relatively less aggressive low grade or secondary gliomas. The HP KIC imaging method can assess activities of two key enzymes in BCAA catabolism, BCAT and BCKDC, and therefore potentially play a crucial role in tumor characterization, drug development and therapeutic decision.

## Conclusion

HP [1-^13^C]KIC is a promising substrate to investigate *in vivo* brain tumor metabolism. KIC can be delivered to intracellular space via MCT and instantly converted to [1-^13^C]leucine by BCAT^24^ or to CO_2_ (and thus HCO_3_^−^) by BCKDC. The detection of BCKDC activity is crucial for assessing BCAA oxidation since it irreversibly catabolizes BCKAs and regulates the BCAA oxidation. Here we demonstrated the feasibility of HP [1-^13^C]KIC to assess BCAT/BCKDC activity in F98 glioma *in vivo* and investigated that leucine oxidation was increased in the tumor as a unique feature of glioma. In addition, we showed the increase of *in vivo* leucine oxidation in F98 glioma as a unique metabolic feature of brain tumor using steady-state infusion [U-^13^C]leucine. Both the *in vivo* HP KIC imaging and the leucine infusion study indicate that KIC catabolism is upregulated through BCAT/BCKDC and further oxidized via the CAC in F98 glioma. To our knowledge, this is the first report of HP [1-^13^C]KIC *in vivo* imaging in rat glioma model. Besides glioma and other cancer applications, we expect that this technique can be utilized to investigate BCAT/BCKDC-associated pathological features in multiple other diseases with altered BCAA metabolism such as traumatic brain injury^45^.

## Methods

### GBM animal model and tumor implantation

F98 glioma cell line was used for both *in vitro* and *in vivo* studies. It is histologically classified as an anaplastic glioma, which is pathologically undifferentiated malignant glioma, and the characteristics of tumor growth are similar with human GBM in overexpression of the EGFR^28^. The F98 glioma cells (CRL-2397^TM^) were obtained from ATCC (Manassas, VA, USA). The cells were cultured at 37 °C in a monolayer using Dulbecco’s Modified Eagle Medium (DMEM) with 10% fetal bovine serum (FBS) and streptomycin with 5% CO_2_^29^. The cells were harvested at 85~90% confluency. Approximately 1 × 10^4^ cells were prepared for each animal. After a Trypan blue exclusion test for cell viability, the cells were implanted into the right striatum of male Fisher rats (n = 12, body weight = 240 ± 10 g). The tumor implantation was performed at the Neuro-Models Core Facility of the University of Texas Southwestern Medical Center. The growth of tumors was confirmed after 15–18 days of implantation by T_2_-weighted and CE T_1_-weighted proton MRI. The tumor regions identified by postmortem H&E stain was better matched to the hyperintense regions of the CE T_1_-weighted-MRI rather than those of T_2_-weighted images (Fig. S1).

When the tumors were established the animals were either injected with HP [1–^13^C]KIC for MRSI or infused with [U-^13^C]leucine for isotopomer analysis. Tumor volumes were estimated as 93.5 ± 10.3 mm^3^ by measuring hyperintense regions in a 2D T_2_-weighted MRI using OsiriX dicom viewer. Tumor sizes were further confirmed by a 2D CE T_1_-weighted MRI (50.0 ± 26.5 mm^3^). Immediately after the imaging or infusion studies, the glioma and NAB tissues were collected for enzyme assays, histology or isotopomer analysis. For comparison, healthy male Fisher rats (n = 3, body weight = 252 ± 10 g) were also examined. All the animal protocols were approved by the local Institutional Animal Care and Use Committee and all experiments were performed in accordance with relevant guidelines and regulations.

### Hyperpolarization study of [1-^13^C] α-ketoisocaproic acid

[1-^13^C] α-ketoisocaproic acid (KIC) was prepared from [1-^13^C] α-ketoisocaproic acid sodium salt (Cambridge Isotope Laboratories, Andover, MA, USA) by dissolving in 1-M HCl (pH < 1) and extracting the aqueous layer with diethyl ether. The combined organic layer was dried over anhydrous sodium sulfate, filtered and evaporated to afford [1-^13^C]KIC (7 M, colorless oil, purity >95%, 94% yield)^25^. For hyperpolarization, a 70-μL sample of the [1-^13^C]KIC mixed with 11-mM trityl radical (OX063, Oxford Instruments Molecular Biotools Ltd, Oxfordshire, UK) was placed in a sample vial, which was then assembled with a research fluid path (GE Healthcare, Waukesha, WI, USA) containing 16 mL of dissolution media (0.1 g/L Na_2_EDTA) in the dissolution syringe. After placed in a SPINlab^®^ polarizer (GE Healthcare), the sample was polarized at ~0.8 K of temperature in a 5 T magnet by irradiating microwaves at a frequency of 139.93 GHz. The polarized sample (3 - 4 hrs) was dissolved and mixed with neutralization media (0.72 M NaOH, 0.4 M Trizma and 0.1 g/L Na_2_EDTA), resulting in 6.5–7.0 mL of 80-mM [1-^13^C]KIC solution (pH 7.4–8.2).

### Hyperpolarized ^13^C MRS study of F98 cell

HP ^13^C MR cell studies were performed on a clinical GE 3 T Discovery 750 W MR scanner (∅_bore_ = 70 cm) and the GE SPINlab™. F98 cells were cultured as described above and collected (1.1 ± 0.1 × 10^8^, n = 3) and washed with PBS twice. The cells suspended in 2 mL of DMEM media were placed in a Falcon^TM^ 50 mL centrifuge tube at 37 °C, and 2 mL of HP [1-^13^C]KIC was injected into the tube within 15–18 sec following the dissolution. Dynamic and time-average ^13^C spectra were acquired using a custom-made ^13^C transmit/receive surface coil (∅_inner_ = 28 mm).

### MR Imaging protocol

*In vivo* imaging studies were also carried out at the clinical GE 3 T MR scanner. For ^1^H imaging, a quadrature birdcage volume RF coil (∅_inner_ = 80 mm) was used for both RF excitation and data acquisition. After locating the rat brain at the center of the birdcage coil using a 3-plane fast gradient-recalled echo (FGRE) sequence, high-resolution axial brain images were acquired using a dual-echo T_2_-weighted fast spin echo (FSE; echo times [TEs] = 11.3 msec/64.0 msec, TR = 5,000 msec, 7–13 slices, field of view [FOV] = 96 × 96 mm^2^, matrix size = 256 × 192, slice thickness = 2 mm, echo train length = 8). B_0_ field inhomogeneity over the brain region was minimized using a single voxel point-resolved spectroscopy (PRESS) sequence by adjusting linear shim currents. For ^13^C MRSI, a 2D spiral chemical shift imaging pulse sequence described in previous study^29^ using the ^13^C surface coil with a variable flip angle scheme^46^, ${\theta }_{n}={\tan }^{-1}(\frac{1}{\sqrt{4-n}}),\,n=1,\,\ldots ,\,4$, was used to acquire single time-point metabolite maps of rat brain (four spatial interleaves of spiral k-space readout, spectral bandwidth = 210.8 Hz, #echoes = 64, FOV = 50 × 50 mm^2^, matrix size = 16 × 16, slice thickness = 7.7 mm, acquisition time = 1.5 sec) following an intravenous injection of 80-mM HP [1-^13^C]KIC (injection-to-scan time = 25 sec, 1 mmol/kg body weight up to 4.0 mL). Finally, a CE T_1_-weighted spin echo (SE) images were acquired at the end of each imaging session (TE = 12 msec, TR = 700 msec, 96 × 96 mm^2^, 7–13 slices, matrix size = 256 × 192, slice thickness = 2 mm).

### Image reconstruction

The HP ^13^C MRSI were reconstructed for individual peak similarly as the described in previous study^30^. After a 5-Hz Gaussian line broadening and 4-fold zero-fillings along the time domain, an inverse FFT was applied to the raw data. Prior to a spatial apodization (hanning function) and a zero-filling (4x) along each spatial domain, chemical shift artifact was removed by correcting the off-resonance and aliasing pattern of each peak (spectral tomosynthesis), followed by a gridding onto the Cartesian coordinate and a spatial 2D inverse FFT.

Metabolic maps of HP [1-^13^C]KIC, [1-^13^C]leucine, and H^13^CO_3_^−^ were produced by integrating the signal around each peak in absorption mode. The metabolic images were normalized by the maximum signal. Regions of interest (ROIs) of tumor and NAB were drawn manually to calculate the signal intensities for the metabolites. All the ^13^C data were reconstructed and analyzed using MATLAB (Mathworks, Natick MA, USA).

### Infusion of [U-^13^C]leucine

^13^C isotope tracer infusion studies were performed as previously descried^4^. After 15–18 days from the tumor implantation, rats were fasted (n = 3) for 15 hrs prior to leucine infusion (U-^13^C; 99%, Sigma-Aldrich Isotec). After a tail vein catheterization under anesthesia (<10 min), all the rats stayed awake during the infusion. Each infusion started with a bolus injection of [U-^13^C]leucine (0.25 mg/g per body weight in 4 mL saline solution over 1 min), continued by a slow infusion (0.0069 mg/g of body weight/min in saline solution at 4.8 mL/hr for 150 min). Following the completion of the infusion, each animal was fully anesthetized and 4–6 mL of blood sample was collected by cardiac puncture. After decapitation, the whole brain was harvested and sliced to 2-mm coronal sections using brain matrices (Ted Pella, Inc., Redding CA, USA). Tumor (112.2 ± 24 mg) and contralateral NAB tissues (92.0 ± 10.8 mg) were rapidly collected from the brain slices, immediately frozen by liquid nitrogen and stored at −80 °C. The quantification of ^13^C fractional enrichment on glutamate methods are in supporting material.

### Statistical analysis

Statistical significance between tumor and contralateral NAB was evaluated by a paired t-test (α = 0.05, one-tailed) using GraphPad Prism version 7.0 (GraphPad Software, Inc., La Jolla CA, USA). Unpaired t-tests were used for comparison between tumor-bearing rats and healthy controls. All the data were presented as mean ± standard deviation.

## Supplementary information

Supplementary Information
