# Supplementary material for: In vivo assessment of increased oxidation of branched-chain amino acids in glioblastoma
Source: Sci Rep. 2019 Jan 23;9:340. doi: 10.1038/s41598-018-37390-0 (PMC6344513; doi:10.1038/s41598-018-37390-0)
Supplement: Supplementary file 1 — Supplementary Information [file 41598_2018_37390_MOESM1_ESM.pdf]

Title: *In vivo* assessment of increased oxidation of branched-chain amino acids in glioblastoma

Authors: Eul Hyun Suh, Edward P. Hackett, R. Max Wynn, David T. Chuang, Bo Zhang, Weibo Luo, A. Dean Sherry, Jae Mo Park

Supplementary Information

- **Supplementary Methods**
- **Supplementary Figures**
- **References**

Supplementary Methods

Quantification of ^{13}C fractional enrichment on glutamate

Quantitative analysis about the downstream metabolic products of [U-^{13}C]leucine was performed using the tissue samples from the infusion study. ^{13}C NMR spectra of PCA tissue extracts of glioma and NAB were compared. [U-^{13}C]leucine was oxidized to [1,2-$^{13}C_2$]acetyl-CoA and [2-^{13}C]acetyl-CoA, which were metabolized into [4,5-$^{13}C_2$]glutamate and [4-^{13}C]glutamate through CAC[1]. Infused ^{13}C-leucine can result in ^{13}C singlets, doublets, or multiples of [4-^{13}C]glutamate, depending on metabolic turnover. Mostly, the singlet of C4 glutamate (C4S) and doublets of glutamate (C4D) were shown in our ^{13}C leucine infusion study. The ^{13}C enrichment of C4 glutamate in brain tissue were determined by C4 glutamate doublet (C4D, $^{1}J_{45}$ = 51.0 Hz) and C4 glutamate singlet (C4S) of the corresponding ^{13}C resonance at 34.4 ppm. However, this singlet of glutamate (C4S) includes natural abundance level (1.1%) of ^{13}C isotope of intrinsic glutamate pool in addition to the singlets resulting from metabolic conversion of [U-^{13}C]leucine to a ^{13}C-enrichment glutamate. To exclude the contribution of natural abundance level, the total concentration of glutamate was measured using ^{1}H NMR and an internal reference of 1-mM DSSd_6. The amount of ^{13}C incorporation into glutamate C4S and C4D was calculated from the ratio of peak area of C4S or C4D over the peak area of the 1-mM DSSd_6 and was presented in μmol/g unit after normalization by wet tissue weight. Therefore, the portion of ^{13}C incorporated in C4 glutamate can be obtained by (β+γ)/α. (**Figure 3d**.)

$\alpha = \frac{\int 1H\ NMR\ of\ C4}{\int Dssd6}$, where α is the total glutamate concentration in the tissue sample.

$\beta = \frac{\int 13C\ NMR\ of\ C4S}{\int Dssd6}$ = [C4S] - 0.01α, where [C4S] is ^{13}C NMR integration of C4S glutamate.

$\gamma = \frac{\int 13C\ NMR\ of\ C4D}{\int Dssd6}$ = [C4D], where [C4D] is ^{13}C NMR integration of doublet of glutamate C4.

Lactate and leucine concentration in brain tissue via ^{1}H NMR

Lactate and leucine concentration of glioma and contralateral NAB brain tissues were determined from the ratio of the area of DSSd_6 (**Figure S2**). Chemical shifts were referred to internal DSSd_6 at 0 ppm, which was used an internal reference and concentration standard. The total leucine plasma level was within the physiological condition during the infusion (91.2 μmol/L in tumor-bearing rats, 152.6 μmol/L in healthy control)[2].

Histology

Paraffin-embedded glioma and H&E staining were obtained from tumor-bearing brain slices by the Division of Molecular Pathology Core Facility at University of Texas Southwestern Medical Center (**Figure S1**).

Radiochemical assay for BCKDC activity

Flash frozen tumor-appearing brain tissue (117.5 ± 32 mg, n = 4) and contralateral NAB tissue (112 ± 21.7 mg, n = 5) were thawed and manually homogenized in ice-cold glass tissue homogenizer containing 1 mL of homogenizing buffer. The homogenizer buffer was composited of 30 mM KPi (pH 7.5), 3 mM EDTA, 5 mM DTT, 3% fetal bovine serum, 5% triton X-100, 1 μM leupeptin, 50 mM NaF, 2.0 mM DCA, and 1.0 mM S-CPP. The tissue homogenate was transferred to an ice-cold 10 mL polycarbonate tube and centrifuged at 25,000 G for 10 min to spin down tissue debris. The supernatant was transferred to a new 10 mL tube and centrifuged at 40,000 G for 90 min to pellet all the branched-chain α-ketoacid dehydrogenase complex (BCKDC). The supernatant was removed and the BCKD containing pellets were resuspended into 1 mL dilution buffer composed of 50 mM HPEPS (pH 7.5), 0.5 mM DTT, 0.1 % Triton X-100, 3 % fetal bovine serum, and 1 μM leupeptin no ice. Samples (50 μL) were placed in 24-well assay plates containing 295 μL of assay buffer composite with 30 mM KPi (pH 7.5), 0.4 mM CoA, 3 mM NAD^+, 5% fetal bovine serum, 2 mM thiamine diphosphate, 2 mM $MgCl_2$, and 42 μg/ml of human E3. 25 μL of [1-^{14}C]KIC substrate (0.5 mM in 25 μL; specific radioactivity: 1,000 cpm/nmol) was added to each well and placed bridge per well containing 2 M NaOH soaked filter wicks. Assay plates were sealed with clear Mylar adhesive film and incubated at 37 °C for 30 min to begin BCKDC activity assay. The plates were cooled down on ice for 10 min. 50 μL of trichloroacetic acid solution (20%) was added to each well to stop the assay reaction. Assay plates were further incubated at 37 °C for 45 min to completely inactivate the BCKDC. The amount of carbon dioxide ($^{14}CO_2$), trapped on 2M NaOH soaked filter wicks, was measured in a liquid scintillation counter[3,4]. Protein concentration of each sample was measured by a Bradford assay with BSA as a standard. BCKDC enzymatic activity was measured as nmol CO_2/min/g tissue.

Spectrophotometric BCAT activity assay

Flash frozen tumor tissue and contralateral NAB tissue were thawed and homogenized as described in the previous section (n = 3 each for tumor and NAB tissue). Spectrophotometric BCAT activity assay was performed from the rat brain tissues as described in Ref 5. Briefly, 20 U/ml L-lactic dehydrogenase (from rabbit muscle, Sigma-Aldrich) and 10 U/ml glutamic-pyruvic transaminase (from porcine heart, Sigma-Aldrich) were prepared in 100 mM potassium phosphate (pH 7.4). Stock solution of the following substrates and enzymatic cofactors was prepared in deionized water: 42 mM KIC, 4 mM NADH, and 2 mM pyridoxal 5'-phosphate hydrate). All the assay solution was kept ice-cold. 100 mL of assay buffer solution containing 0.2M Tris, 0.6 M L-glutamic acid, and 1.6 M L-alanine (pH 8.3) and 10 µL of substrate stock solution was placed in 96-well assay plates. 2 µL of tissue homogenate and 48 µL of deionized water were added to each well, yielding a final solution of 200 µL per well. Absorbance of 334 nm wavelength at 37 °C was monitored every 30 sec for 30 min. Specific activity (U/gram protein) was calculated from protein concentration measured via Bradford assay.

Immunoblot

Tissues were lysed in lysis buffer (50 mM Tris-HCl, pH 7.4, 150 mM NaCl, 1% Triton-X100, and protease inhibitor cocktail) and were homogenized (BioSpec-985370 Tissue-Tearor Homogenizer) on ice, followed by sonication on ice. Equal amounts of lysates were fractionated by SDS-PAGE and subjected to immunoblot assays with the following antibodies: BCAT1 (Proteintech, 13640-1-AP), BCAT2 (Proteintech, 16417-1-AP), actin (Proteintech, 66009-1-Ig), BCKDE1A (Bethyl, A303-790A), and BCKDK (Abcam, ab151297)

Supplementary Figures

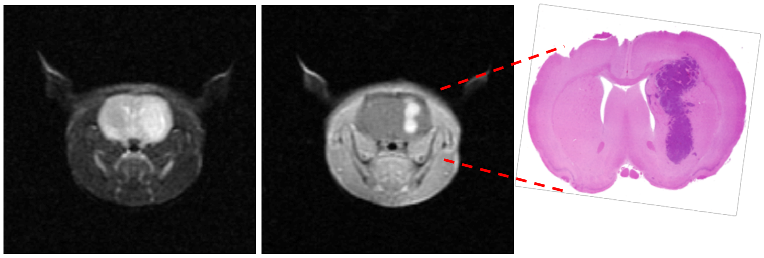

Figure S1. T$_2$-weighted and contrast-enhanced T$_1$-weighted proton MRI from an axial slice of rat brain 15~18 days after F98 cell implantation. H&E histology confirmed the tumor.

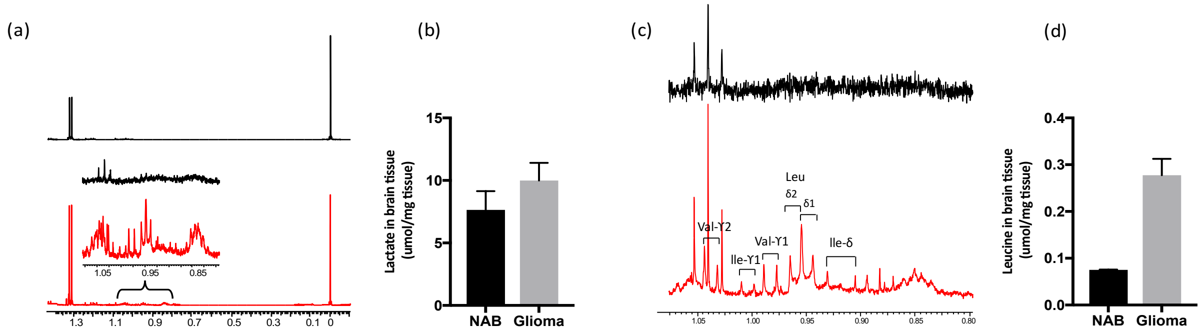

Figure S2. ^{1}H NMR spectra acquired from NAB (black spectrum) and glioma (red spectrum) after [U-^{13}C]leucine infusion. Elevated lactate (a) and leucine uptake (c) were measured in glioma compared to NAB (b,d).

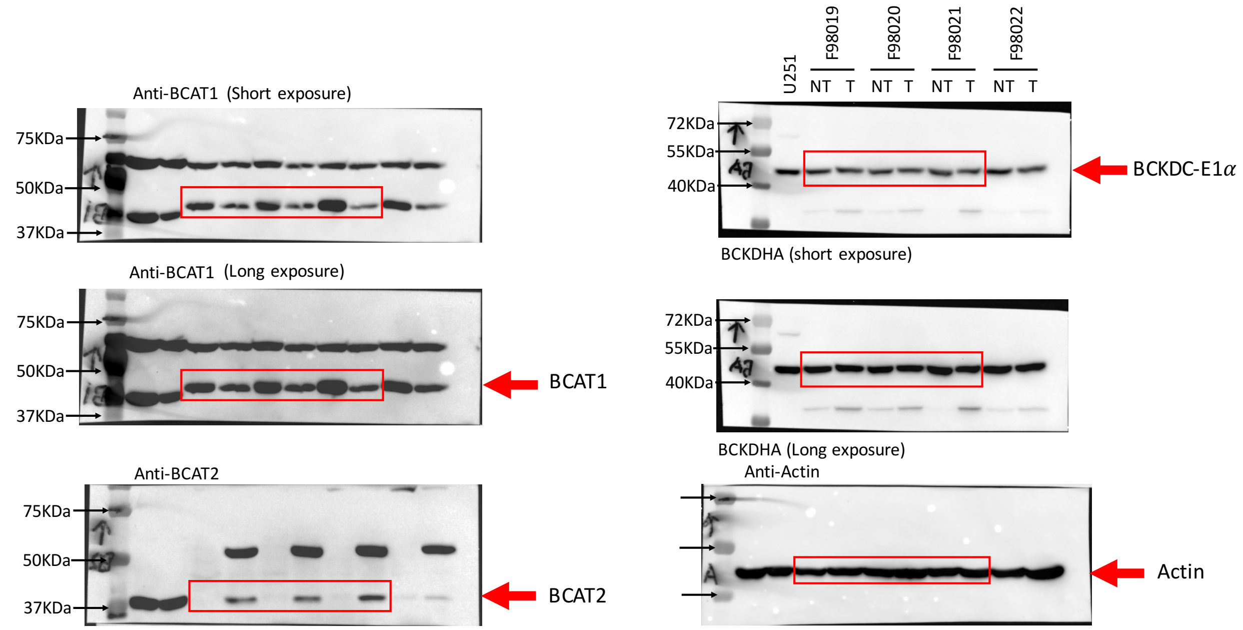

Figure S3. Unprocessed western blots for Supplementary **Figure 4b**.

References

1. Bixel, M.G., Engelmann, J., Willker, W., Hamprecht, B. & Leibfritz, D. Metabolism of [U-(13)C]leucine in cultured astroglial cells. *Neurochemical research* **29**, 2057-2067 (2004).
2. Holecek, M., Siman, P., Vodenicarovova, M. & Kandar, R. Alterations in protein and amino acid metabolism in rats fed a branched-chain amino acid- or leucine-enriched diet during postprandial and postabsorptive states. *Nutrition & metabolism* **13**, 12 (2016).
3. Chuang, D.T. & Cox, R.P. Enzyme assays with mutant cell lines of maple syrup urine disease. *Methods in Enzymology* **166**, 135-146 (1988).
4. Tso, S.-C.*, et al.* Structure-based design and mechanisms of allosteric inhibitors for mitochondrial branched-chain α-ketoacid dehydrogenase kinase. *Proceedings of the National Academy of Sciences* **110**, 9728-9733 (2013).
5. Schadewaldt, P. & Adelmeyer, F. Coupled Enzymatic Assay for Estimation of Branched-Chainl-Amino Acid Aminotransferase Activity with 2-Oxo Acid Substrates. *Analytical Biochemistry* **238**, 65-71 (1996).
